# Continuous Kalman Estimation Method for Finger Kinematics Tracking from Surface Electromyography

**DOI:** 10.34133/cbsystems.0094

**Published:** 2024-05-15

**Authors:** Haoshi Zhang, Boxing Peng, Lan Tian, Oluwarotimi Williams Samuel, Guanglin Li

**Affiliations:** ^1^ CAS Key Laboratory of Human-Machine Intelligence-Synergy Systems, Shenzhen Institutes of Advanced Technology (SIAT), Chinese Academy of Sciences (CAS), Shenzhen 518055, China.; ^2^ Shenzhen College of Advanced Technology, University of Chinese Academy of Sciences, Shenzhen 518055, China.; ^3^ Shandong Zhongke Advanced Technology Co. Ltd., Jinan 250000, China.; ^4^ School of Computing, University of Derby, Derby DE22 3AW, UK.

## Abstract

Deciphering hand motion intention from surface electromyography (sEMG) encounters challenges posed by the requisites of multiple degrees of freedom (DOFs) and adaptability. Unlike discrete action classification grounded in pattern recognition, the pursuit of continuous kinematics estimation is appreciated for its inherent naturalness and intuitiveness. However, prevailing estimation techniques contend with accuracy limitations and substantial computational demands. Kalman estimation technology, celebrated for its ease of implementation and real-time adaptability, finds extensive application across diverse domains. This study introduces a continuous Kalman estimation method, leveraging a system model with sEMG and joint angles as inputs and outputs. Facilitated by model parameter training methods, the approach deduces multiple DOF finger kinematics simultaneously. The method’s efficacy is validated using a publicly accessible database, yielding a correlation coefficient (CC) of 0.73. With over 45,000 windows for training Kalman model parameters, the average computation time remains under 0.01 s. This pilot study amplifies its potential for further exploration and application within the realm of continuous finger motion estimation technology.

## Introduction

With the advancement of robotics technology, the application of robotic hands has expanded considerably, encompassing diverse fields including search and rescue operations, space teleoperation, service industries, industrial applications, and artificial prosthetics. In the pursuit of achieving more seamless human–machine integration, substantial endeavors have been directed toward emulating human-hand functionalities based on the user’s motor intentions [1]. With advantages of simple acquisition process and being noninvasive, surface electromyography (sEMG), which reflects human neuromuscular activities, serves as a direct source for decoding human motor intentions and has gained widespread adoption for enabling dexterous control of robotic hands globally [2,3].

Presently, technologies centered around decoding motion intentions from sEMG primarily gravitate toward two trajectories: pattern recognition and continuous kinematics estimation. The conventional pattern recognition paradigm relies on manually selected features and machine learning algorithms to establish a correspondence between signal features and discrete motion patterns. Prominent machine learning techniques such as linear discriminant analysis (LDA), support vector machine (SVM), and artificial neural networks (ANNs) have showcased high accuracy in offline classification [4]. Deep learning (DL) approaches have also emerged to tackle more intricate motion classes [5]. While pattern recognition techniques uniformly treat all motions within the target space, regardless of their complexity or degree of freedom (DOF) count, enabling swift recognition of intricate motions, they are restricted to predefined discrete motion types. Consequently, these methods permit the recognition of only one motion at a time and result in abrupt and unnatural transitions between different motions. Moreover, ensuring high classification accuracy demands repetitive execution of motions, rendering recognition accuracy sensitive to changes in motion execution patterns.

On the other hand, continuous kinematics estimation methodologies revolve around continuous variables, aiming to estimate joint angles or torques across multiple DOFs based on sEMG. These methodologies enable users to achieve proportional control over multi-DOF equipment simultaneously, offering a more intuitive and natural form of device control. Regression models such as linear regression, neural networks, Gaussian process regression, and support vector regression are typically employed to establish connections between signal features and one or more continuous kinematics variables [6]. The concept of proportional control inspired by muscle synergy has been introduced for robotic control involving two or three DOFs [7,8], but its current application has been limited to wrist tracking. Despite various endeavors in different techniques, accurately elucidating the relationship between EMG signals and motion remains challenging, resulting in approximations in current continuous kinematics estimation outcomes.

Discrete-time Kalman estimation offers a simple closed-form recursive solution for estimating linear discrete-time dynamic systems. Renowned for its low computational load and high speed, this method finds widespread applicability in estimation challenges spanning from target tracking to vehicle control. Numerous extended Kalman technologies have been devised to cater to diverse demands across various domains. Wu et al. [9] introduced a method involving derived closed-loop solutions of Kalman equation coefficients based on training datasets. Subsequently, the Kalman decoding technique gained prominence in decoding hand kinematics from neural activity in the motor cortex [10]. Elmohandes et al. [11] delved into the feasibility of tracking two wrist joint angles from EMG signals using the Kalman decoding method. Nonetheless, research investigating the impact of this method on simultaneously tracking multi-DOF finger joint hand kinematics remains limited.

In the quest to enhance the precision of finger kinematics estimation while also reducing computational expenses, this study introduces the Kalman decoding technique for tracking finger kinematics using sEMG. As a pilot study, the feasibility and implications of this approach are examined. The establishment of a continuous Kalman estimation framework for tracking hand joint angles holds the potential to pave the way for integrating various existing Kalman extension techniques into this application domain, thereby propelling the evolution of hand motion estimation.

## Methods

### Database

**Fig. 1.**
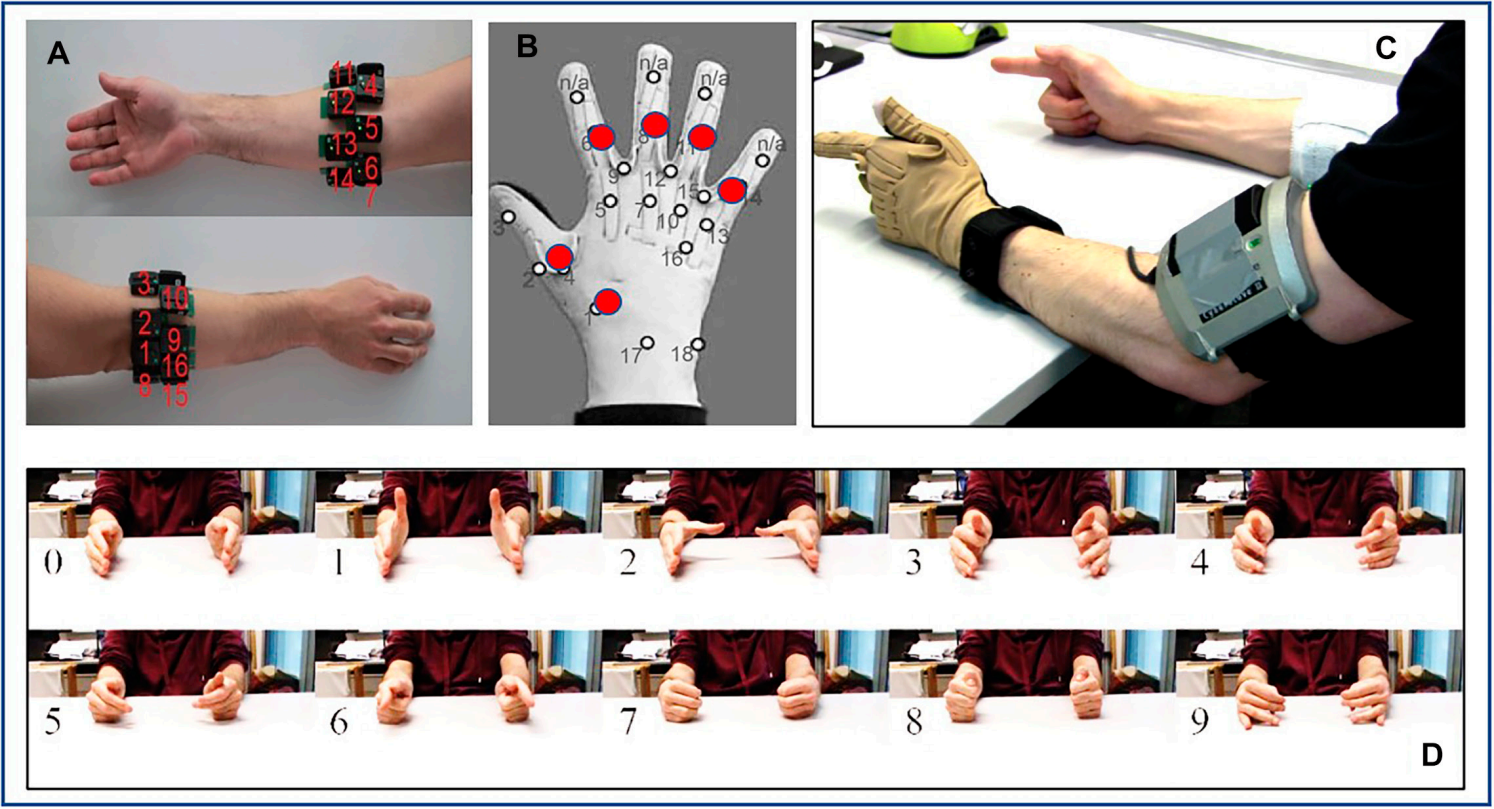
Dataset description. (A) Distribution of 16-sEMG electrodes. (B) Distribution of the estimated six joint angles denoted as red dots. (C) Experimental scene of bilateral mirrored movements. (D) Distinct finger movements included in the experiments.

The effectiveness of the proposed continuous Kalman estimation method for multiple finger kinematics tracking was evaluated using the Ninapro database [12], a publicly accessible repository created to support research in the realms of artificial intelligence robotics and prosthetic hands. Specifically, we employed the eighth database (DB8), designed to cater to the estimation of finger movements from sEMG signals [13]. This comprehensive dataset encompasses a 16-channel EMG signal collection obtained from the right arm using the Delsys Trigno IM Wireless system (Fig. 1A), as well as contralateral hand kinematic data derived from Cyberglove2, an 18-DOF model. The sEMG signals were captured at a rate of 1,111 Hz, while the Cyberglove signals were sampled at 100 Hz. All signals were subsequently upsampled to 2 kHz and synchronized.

In this study, we aimed to estimate the joint angle values of six DOFs, which correspond to the primary active joints engaged in grasping movements: thumb flexion/extension, thumb abduction/adduction, index flexion/extension, middle flexion/extension, ring flexion/extension, and little flexion/extension, as depicted in Fig. 1B. Data collection entailed participants replicating nine distinct finger movements using both hands, leading to bilateral mirrored movements (Fig. 1C and D).

The dataset comprises 12 participants, encompassing 10 able-bodied individuals (subjects 1 to 10) and 2 right-hand transradial amputees (subjects 11 and 12). Each participant contributed three distinct datasets. For assessing the robustness of the proposed Kalman estimation method, the first two datasets (dataset 1 and dataset 2) were utilized to train the model parameters, while the third dataset (dataset 3) was reserved for testing and validating the estimation performance of the model.

### Signal preprocessing

The sEMG signals underwent initial filtration utilizing a Butterworth bandpass filter with a cutoff frequency of 10 to 450 Hz to retain the principal components of the sEMG signal. Subsequently, considering the existence of electromechanical delay (EMD) during sEMG data and motion data collection, where the onset of sEMG signals occurs 30 to 150 ms earlier than the onset of the movements [14,15], window-based feature extraction strategy is employed to mitigate the impact of joint angle data delay on estimation results. A sliding window with window length of 150 ms (300 sample points) and step length of 25 ms (50 sample points) was employed to segment the sEMG and finger joint angle signals into contiguous windows. Following segmentation, the mean absolute value (MAV) was computed for each sEMG window, forming the basis for the construction of the sEMG feature matrix. Similarly, the last one value of the joint angle within each window was used to construct the joint angle feature matrix. Notably, the feature extraction process for both sEMG and finger joint angles was performed individually across the three datasets for each subject.

### Continuous Kalman estimation method

The continuous Kalman estimation method treats vectors of joint angle values across multiple DOFs and sEMG feature vectors as inputs and outputs within a dynamic linear system. This system is characterized by constructing both a system state equation and an observation equation, collectively forming the Kalman estimation model. Once established, this model enables real-time estimation of output vector values based on specific iterative criteria.

#### State equation

The state equation defines the relationship between adjacent hand kinematic states at time *k* + 1 and time *k* (formulated as Eq. 1):

$$x_{k+1}={\mathrm{Ax}}_{k}+w_{k}$$ (1)

Here, $x_{k}={\left[\theta_{1},\theta_{2},\theta_{3},\;\ldots ,\theta_{n}\right]}_{k}^{T}$ signifies the current state vector at time *k*, comprising angle values $\theta_{1}∼\theta_{n}$ corresponding to *n* joint DOFs (*n* = 6 in this study). *A* denotes the state transition coefficient matrix, and $w_{k}$ represents the state noise term assumed to possess a zero mean and normally distributed with covariance matrix $E\left\{w_{k}w_{k}^{T}\right\}=W$.

#### Observation equation

Equation 2 constitutes the observation equation, outlining the connection between the current extracted observation vector $z_{k}$ and the state vector $x_{k}$. The observation vector $z_{k}$ encompasses the current sEMG feature vectors, while the state vector $x_{k}$ is mapped to the observation vector $z_{k}$ through the observation coefficient matrix *H*. The observation noise term $q_{k}$ is similarly assumed to exhibit a zero mean and normally distributed with covariance matrix $E\left\{q_{k}q_{k}^{T}\right\}=Q$.

$$z_{k}={\mathrm{Hx}}_{k}+v_{k}$$ (2)

In instances where *A*, *H*, *W*, and *Q* within the Kalman estimation model are known, the classical Kalman filter iteration method can be applied to sequentially estimate the joint angle state vector $x_{k}$ step by step. Assuming that *A*, *H*, *W*, and *Q* remain constant, Wu et al. proposed a solution to estimate these coefficients from training data through the least squares estimation [13].

$$A=\underset{A}{\arg\;\min}\sum_{k\;=\;1}^{M-1}{\left\|x_{k+1}-{\mathrm{Ax}}_{k}\right\|}^{2}$$ (3)

$$H=\underset{H}{\arg\;\min}\sum_{k\;=\;1}^{M}{\left\|z_{k}-{\mathrm{Hx}}_{k}\right\|}^{2}$$ (4)

Here, *M* represents the number of time steps in the training dataset, signifying *k* = 1, 2,…, *M* for $x_{k}$ and $z_{k}$. Moreover, ‖.‖ denotes the conventional norm. The solutions are expressed as:

$$A=X_{2}X_{1}^{T}{\left(X_{1}X_{1}^{T}\right)}^{-1}$$ (5)

$$H={\mathrm{ZX}}^{T}{\left(XX^{T}\right)}^{-1}$$ (6)

where *X* = [x_1_, x_2_, …, x*_M_*], *X*_1_ = [x_1_, x_2_, …, x_*M* − 1_], *X*_2_ = [x_2_, x_2_, …, x*_M_*], and Z = [z_1_, z_2_, …, z*_M_*]. Subsequently, *W* and *Q* can be estimated using the derived *A* and *H*:

$$W=\left(X_{2}-{\mathrm{AX}}_{1}\right){\left(X_{2}-{\mathrm{AX}}_{1}\right)}^{T}/\left(M-1\right)$$ (7)

$$Q=\left(Z-\mathrm{HX}\right){\left(Z-\mathrm{HX}\right)}^{T}/M$$ (8)

Leveraging the terms mentioned above, the classical Kalman filter algorithm can be employed to predict the current state of the hand joint angle state vector $x_{k}$ from new sEMG feature vector $z_{k}$. This process entails two stages: prediction and update. During the prediction step, a priori estimate ${\hat{x}}_{k}^{-}$ is derived from the previous state vector ${\hat{x}}_{k-1}$, and the error covariance matrix $P_{k}^{-}$ is computed:

$${\hat{x}}_{k}^{-}=A{\hat{x}}_{k-1}$$ (9)

$$P_{k}^{-}={\mathrm{AP}}_{k-1}A^{T}+W$$ (10)

Subsequently, in the update step, the priori estimate ${\hat{x}}_{k}^{-}$ is revised to ${\hat{x}}_{k}$, and the error covariance matrix $P_{k}$ is updated as follows:

$$K_{k}=P_{k}^{-}H^{T}{\left(HP_{k}^{-}H^{T}+Q\right)}^{-1}$$ (11)

$${\hat{x}}_{k}={\hat{x}}_{k}^{-}+K_{k}\left(Z_{k}-H{\hat{x}}_{k}^{-}\right)$$ (12)

$$P_{k}=\left(I-K_{k}H\right)P_{k}^{-}$$ (13)

**Fig. 2.**
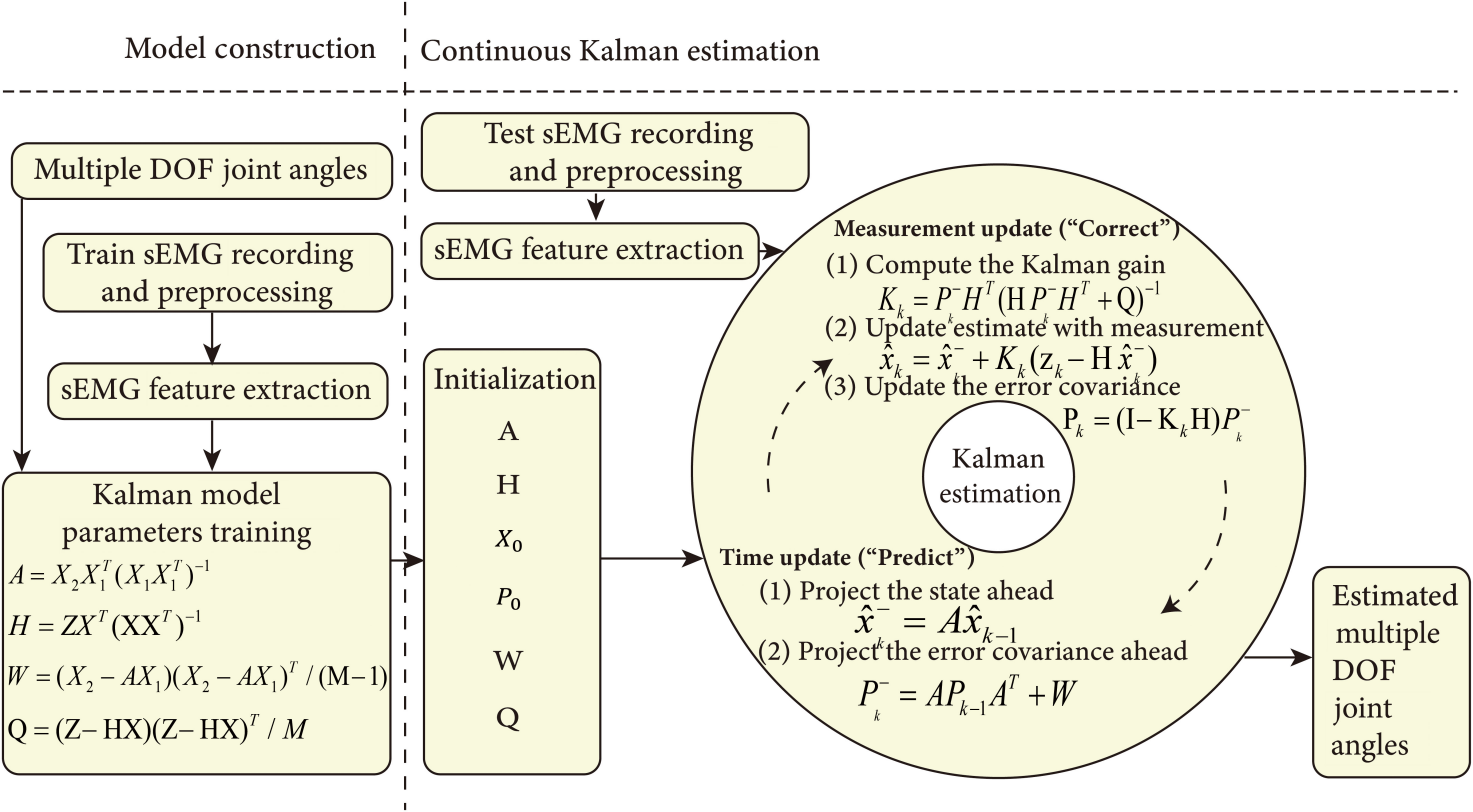
Implementation flowchart of the proposed continuous Kalman estimation method for multiple DOF hand joint angle estimation.

Figure 2 illustrates the comprehensive application flow of the proposed Kalman estimation model. The entire system process is divided into two segments: Kalman model parameter training and real-time Kalman estimation. The training segment corresponds to Eqs. 5 to 8, primarily utilized to acquire model parameters. The testing segment aligns with Eqs. 9 to 13, representing the classic iterative process of Kalman filtering.

### Performance metrics for evaluation

The Pearson correlation coefficient (CC), a standard measure for quantifying the linear relationship between two sequences [16], was employed in this study. Specifically, we calculated the CC between the actual joint angle values $\theta_{n}$ and their corresponding estimated joint angle values ${\hat{\theta }}_{n}$ to assess their level of similarity. The calculation is formulated as follows:

$$\mathrm{CC}=\frac{\sum_{i=1}^{L}\left(\theta -\bar{\theta }\right)\left(\hat{\theta }-\bar{\hat{\theta }}\right)}{\sqrt{\sum_{i=1}^{L}{\left(\theta -\bar{\theta }\right)}^{2}}\sqrt{\sum_{i=1}^{L}{\left(\hat{\theta }-\bar{\hat{\theta }}\right)}^{2}}}$$ (14)

## Results

**Fig. 3.**
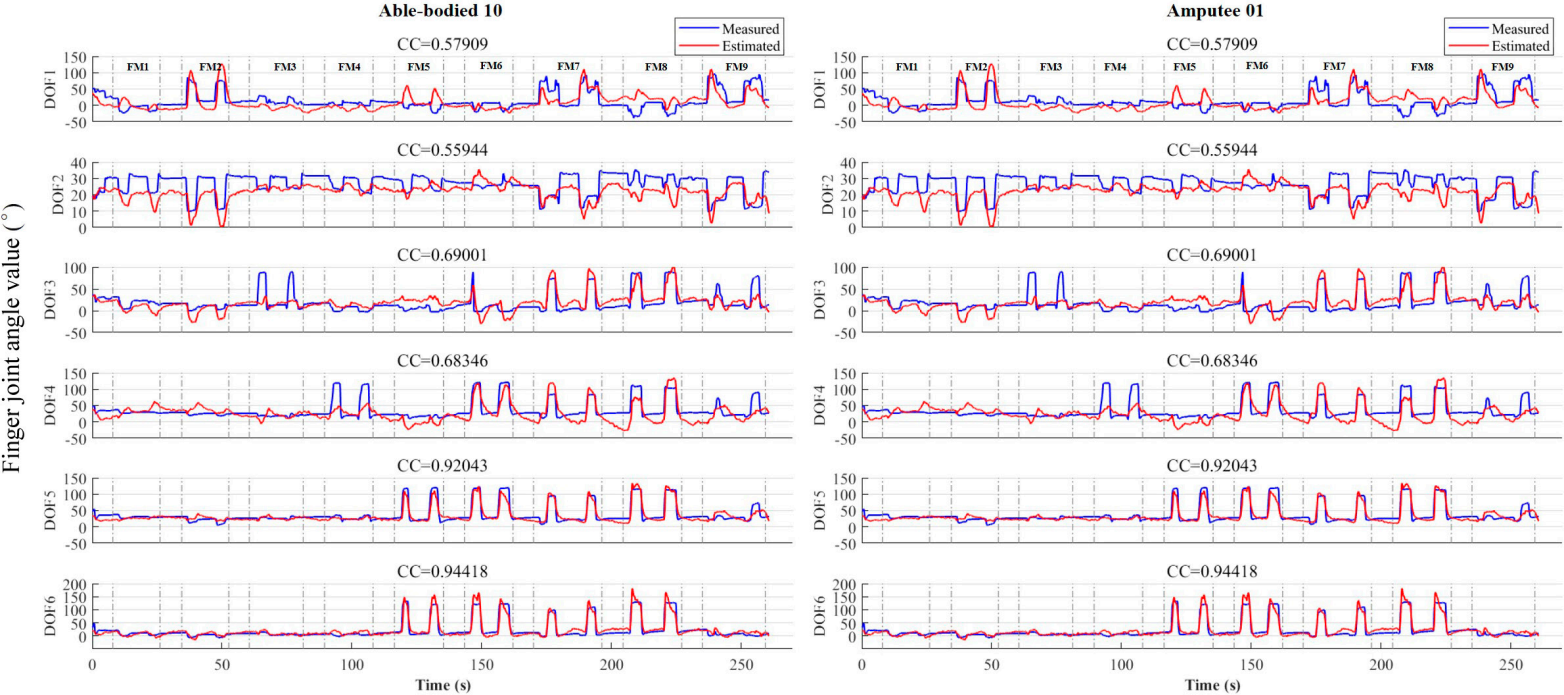
Finger joint angle value estimation. Sample test dataset 3 estimation effects are depicted for all six DOFs of the hand foran able-bodied (left) and an amputee subject (right). FM number denotes the corresponding finger movements.

An illustrative example of the estimated joint angles (depicted in red) juxtaposed with the measured curves (displayed in blue) for both able-bodied and amputee subjects is presented in Fig. 3. The corresponding quantitative evaluation index, the Pearson CC, is displayed on each respective figure. Notably, the results demonstrate the method’s capability to accurately capture substantial joint motion for all DOFs, for both able-bodied and amputee participants, albeit with minor discrepancies. Significantly, DOFs with distinct motion and nonmotion disparities, such as DOF 5 and DOF 6 for the able-bodied subject (Fig. 3A), exhibit precise estimation alignment. The estimated angles closely approximate the true values measured by the data glove, yielding high CC values of 0.92 and 0.94, respectively. Moreover, the Kalman estimation method exhibits swift adaptation to changes in joint angles, evidenced by the prompt and accurate tracking of joint angle variations. This attribute is crucial for achieving real-time estimation accuracy.

**Fig. 4.**
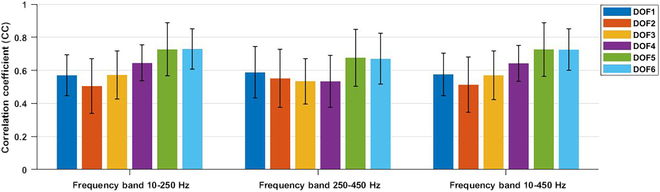
The overall performance of finger joint estimation for each DOF.

The overall performance of finger joint estimation for each DOF is illustrated in Fig. 4, where the CC values are averaged across all subjects under three frequency bands. Notably, DOF 5 and DOF 6 exhibit higher CC values compared to the other DOFs across all three frequency bands, with average CC values of 0.73 ± 0.16 and 0.73 ± 0.13, respectively. Interestingly, the CC values for the same DOFs do not significantly differ among different frequency bands.

**Fig. 5.**
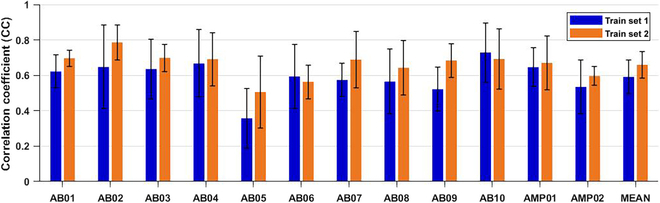
The estimation results for all subjects using different training datasets to compute the model parameters.

The stability of the training scheme in the algorithm was evaluated using different training datasets (dataset 1 and dataset 2) for all 10 able-bodied subjects (subjects 1 to 10) and 2 right-hand transradial amputee participants (subjects 11 and 12). The sEMG features extracted from dataset 3 were then input into the model for joint angle value estimation. The CC between the estimated and real joint angle values recorded by the data glove was computed for each participant. As depicted in Fig. 5, no significant difference in estimation accuracy was observed when using different training datasets. Notably, the estimated results for the two amputees were comparable to those of the able-bodied subjects, highlighting the stability of the method.

In addition to the accuracy of joint angle estimation, computational costs were tested. As indicated in Table, with an average number of windows exceeding 45,000 for training the Kalman model parameters, the average computation time was less than 0.01 s. These results indicate significantly lower computational costs compared to alternative estimation methods.

## Discussion and Conclusion

The realm of hand motion intention decoding faces intricate challenges attributed to the multi-DOF requirements. The ability to accurately perform continuous estimation of finger joint angles holds the potential for achieving simultaneous and proportional control over multi-DOF, offering a more intuitive and natural device control, a notion embraced by numerous researchers [16,17].

Kalman estimation stands as an algorithm employing linear system state equations to optimally estimate the system state via input and output observation data. Given that observation data inherently encompass noise and system interference effects, the optimal estimation process aligns with filtering principles [10]. This technique can effectively estimate dynamic system states from measured data enveloped in measurement noise, particularly when measurement variances are known. Given its ease of implementation in computer programming and real-time on-the-fly data processing and updates, Kalman estimation has gained extensive traction across various domains, including communication, navigation, guidance, and control [18,19].

Despite the complexity of human motion control within dynamic systems, linear relationships manifest in many facets. Researchers have endeavored to simplify control processes using diverse linear systems. In this study, the control of upper limb human motion was conceptualized as a linear dynamic system, wherein joint angles represented system state variables, and sEMG readings functioned as the observed system variables. Notably, when the observed variable, i.e., the sEMG signal, is available, classical Kalman estimation technology, renowned for its performance across diverse applications, could be aptly applied to estimate the joint angles of the state variable.

In our study, a continuous Kalman estimation method for estimating multiple DOF hand joint angles from sEMG was developed. We conducted performance evaluations by using publicly available datasets, and the results affirm the method’s feasibility and stability across diverse training sets. With a CC of 0.73, the Kalman estimation method demonstrated estimation accuracy comparable to large-scale model studies such as DL and neural networks [13,20–22], even without optimization. In addition to the precision of joint angle estimation, the training time of the model is a critical consideration. Given that current estimation techniques have not achieved complete generalization, varying electrode positions can impact estimation results. To optimize estimation accuracy, training the model before for each time utilization is essential. Excessive training times, as seen in many DL techniques requiring several tens of minutes [20], significantly limit the practical application of the method. We conducted an assessment of the training computational cost, revealing that the window average count for training Kalman model parameters exceeds 45,000, with an average computation time of less than 0.01 s, as detailed in Table. These results signify a notable reduction in computational costs compared to various DL estimation methods. The continuous Kalman estimation method emerges as a potent approach, significantly reducing model training time while upholding equivalent estimation accuracy. Moreover, the construction of the Kalman model framework empowers us to explore diverse extensions and variant algorithms of the Kalman filtering technique in our future research, aimed at real-time joint angle estimation. This presents a promising avenue for further improving estimation performance.

**Table.** Computational time to train Kalman estimation model for all subjects under two datasets

	Train dataset 1		Train dataset 2	
Subjects	Window number	Train time (s)	Window number	Train time (s)
S1	45,845	0.0116	46,264	0.0109
S2	45,554	0.0083	46,385	0.0088
S3	45,389	0.0090	46,093	0.0093
S4	45,405	0.0103	46,366	0.0088
S5	41,625	0.0162	46,322	0.0101
S6	45,613	0.0090	46,931	0.0084
S7	45,267	0.0102	46,259	0.0100
S8	45,097	0.0098	45,912	0.0089
S9	45,181	0.0087	46,106	0.0091
S10	45,319	0.0088	46,095	0.0109
S11	45,527	0.0072	46,250	0.0103
S12	45,455	0.0108	45,873	0.0089
Mean	45,106	0.0100	46,238	0.0095

It is important to recognize that Kalman filtering, as an estimation technique, necessitates knowledge of the system’s state equation. By fine-tuning noise parameters, the method approximates the real-world scenario, resulting in stable prediction outcomes. In this study, as depicted in Fig. 2 and Eqs. 3 to 8, the linear system’s state parameters and noise coefficients were acquired through a training process. The accuracy of joint angle estimation using the Kalman estimation model largely hinges on the precise representation of model parameters within the system. Hence, obtaining appropriate model parameters constitutes a pivotal aspect for future research endeavors. Just as various techniques exist to train classifier parameters in pattern recognition domains, diverse approaches can be contemplated for acquiring Kalman model parameters. Examples encompass neural networks and DL, opening avenues for parameter training.

Moreover, it is essential to highlight that the choice of sEMG features can significantly impact the accuracy of the estimated results. In our one previous study [23], a combination of four classical time-domain features [MAV, number of zero crossings (ZCs), waveform length (WL), and number of slope sign changes (SCs)] was employed for joint angle estimation in each window. In this study, only MAV features were utilized. Comparing the results, we observed that increasing the number of features may lead to a certain level of estimation accuracy for some DOFs or for some subjects. Nevertheless, when considering the overall average estimation accuracy, the results obtained using only the MAV feature were not significantly inferior to those using four features. It is important to recognize that an increase in the number of features does not necessarily equate to better performance. The effectiveness of features and their correlation with the estimated quantities need to be carefully considered. The extraction of feature vectors from sEMG signals played a pivotal role in managing signal dimensionality. Identifying feature vectors with enhanced correlation to the joint angle state vector, thus better shaping the system model, represents a promising avenue for expanding the Kalman decoding method toward motion prediction.

The current pilot study delved into the application of the Kalman decoding method for continuous joint angle estimation. In fact, by encoding motion types with corresponding relative joint angle values and incorporating a judgment step subsequent to predicted state outputs, the Kalman decoding method can extend to action pattern classification. Through apt modeling, it may even offer innovative solutions in the realm of integrated action judgments.

## Data Availability

All data needed to evaluate the conclusions in the paper are publicly available at DB8 of Ninapro database. You can get them via: https://ninapro.hevs.ch/instructions/DB8.html.
